# Fertilization-Defective Gametophytic Mutant Screening: A Novel Approach

**DOI:** 10.3389/fpls.2020.00967

**Published:** 2020-06-30

**Authors:** Prakash Babu Adhikari, Xiaoyan Liu, Ryushiro D. Kasahara

**Affiliations:** ^1^School of Life Sciences, Fujian Agriculture and Forestry University, Fuzhou, China; ^2^Horticultural Plant Biology and Metabolomics Center (HBMC), Fujian Agriculture and Forestry University, Fuzhou, China

**Keywords:** fertilization recovery, gametophytic mutant, segregation distortion, female gametophyte, vanillin staining

## Abstract

Gametophytic mutants share very small proportion of the total mutants generated by any mutagenic approach; even rarer are the fertilization-defective gametophytic mutants. They require an efficient and targeted strategy instead of ‘brute force’ screening approach. The classical gametophyte mutant screening method, mainly based on the segregation distortion, can distinguish gametophytic mutants from the others. However, the mutants pooled after the screening constitute both fertilization-defective and developmental-defective gametophytic mutants. Until recently, there has not been any straightforward way to screen the former from the latter. Additionally, most of the mutations affecting both gametes are lost during the screening process. The novel gametophyte screening approach tends to circumvent those shortcomings. This review discusses on the classical approach of gametophytic mutant screening and focuses on the novel approach on distinguishing fertilization-/developmental-defective gametophytic mutants (both male and female). It offers an empirical basis of screening such mutants by taking in the consideration of earlier studies on fertilization failure, initiation of seed coat formation, and fertilization recovery system in plants.

## Introduction

Fertilization is an important event that alternates the gametophytic generation to sporophytic one in plants. Fruit and seed development is completely halted in its absence (Vivian-Smith et al., 2001). While there are some mutants/species which can produce seeds without fertilization, such occurrences are rare in nature. To date, only few plant-fertilization related genes have been identified (Adhikari et al., 2020). Studies in *Arabidopsis* suggest that a distinct group of genes (known as imprinted genes) is essential for the successful seed development. Their active allele is restricted either to the maternal or to the paternal line. Moreover, their expression is mostly limited to the endosperm (and zygote, to some extent) after fertilization (Hsieh et al., 2011; Satyaki and Gehring, 2017). Being directly linked to food crop production, identification of the genetic players and understanding the underlying molecular network during fertilization process is crucial. Such knowledge helps plant breeders find and determine the potentials and limitations of increasing crop production by intervening it at the molecular level. The mutants discussed in this article represent the fully penetrant hemizygous mutants (heterozygous for mutant allele) unless stated otherwise.

## Classical Gametophytic Mutant Screening by Tagging and Chasing

The study of gametophytic mutation has long been the field of interest among plant biologists. However, the lack of proper tools and techniques has been the major bottleneck. Generation and screening of the gametophytic mutants used to be cumbersome until Feldmann et al. (1997) observed the exceptional (non-Mendelian) segregation ratio of a selection marker (kan^R^) in few T-DNA insertional mutants. Such segregation pattern was later termed as ‘segregation distortion’ and has since been regularly used to screen the gametophytic mutants (Christensen et al., 1998; Grossniklaus et al., 1998; Howden et al., 1998; Grini et al., 1999; Boavida et al., 2009). A major drawback of this approach however, is the absolute requirement of selection/reference marker in the mutants.

**Figure 1:**
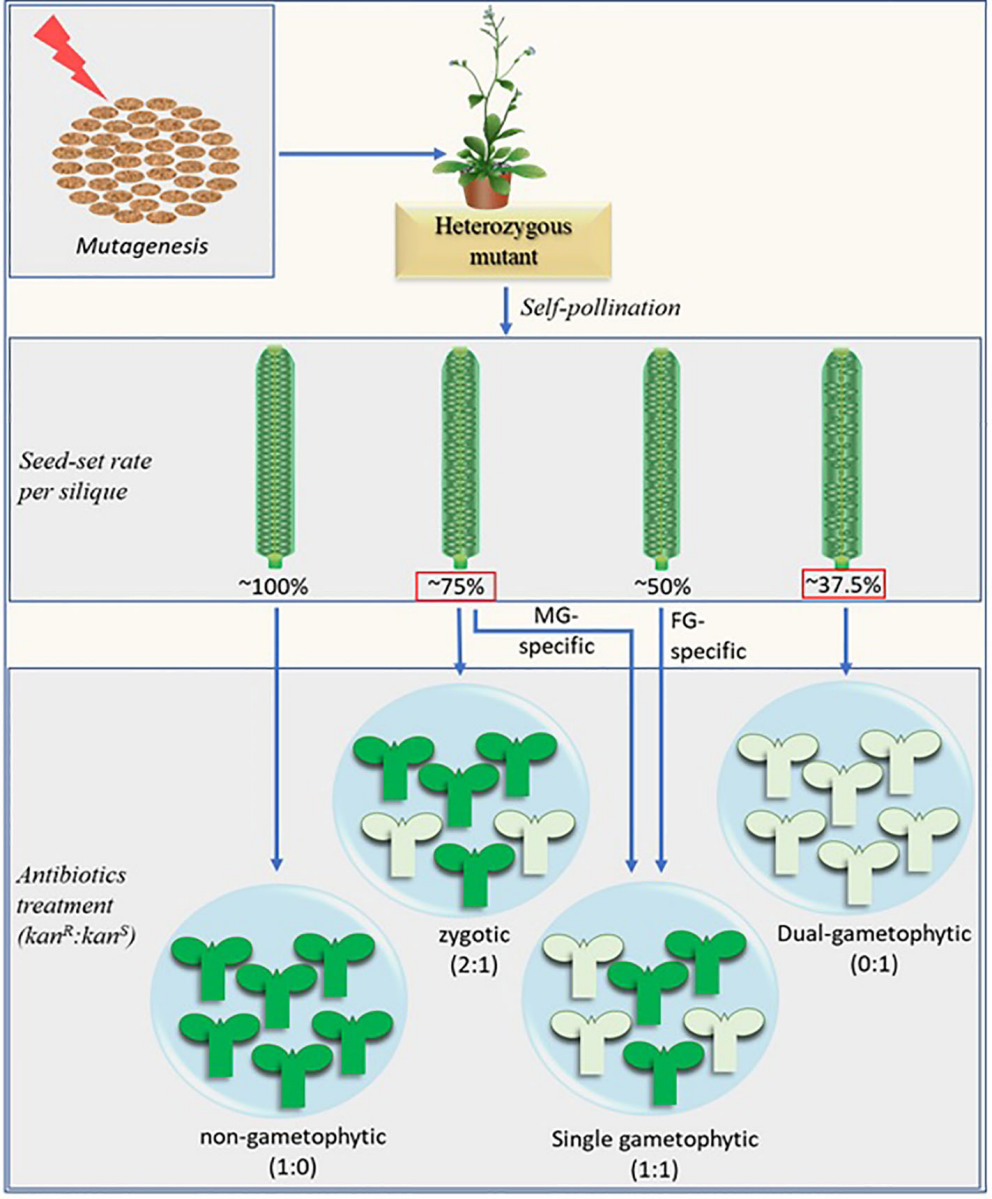
Schematic representation of the modified screening method combined with the potential distortion in seed-set rate brought about by the fertilization recovery system (Beale et al., 2012; Kasahara et al., 2012). The numerical figures represent the theoretically possible maximum values. The fully penetrant dual-gametophytic fertilization defective mutants (with defect in both MG and FG) may show the lowest seed-set (37.5%). None of their progenies will carry the selection marker. The MG-specific and zygotic mutants, on the other hand, may show similar seed set rates (~75%), but their progenies reveal different selection marker segregation ratio (1:1 and 2:1 respectively). The seed-set rates in red squares are derived by considering the fertilization recovery system which is crucial for MG-specific and dual-gametophytic mutant identification via novel screening method. The MG-specific mutants mentioned here are equivalent to sperm cell-specific mutants. Aborted ovules are depicted by the smaller oval structures in the siliques, marker-positive and marker-negative progenies are represented by the green and pale-green seedling structures respectively. Kanamycin (kan) is shown as a representative selection marker.

The classical gametophytic-mutant screening process essentially consists of two consecutive steps after mutagenesis. First, mutants are screened based on their reduced seed-set. In the subsequent step, potential gametophytic mutants are screened based on the distorted segregation of the selection marker in their progenies. A fully penetrant hemizygous gametophytic mutant produces progenies at a 1:1 (marker positive: marker negative) ratio. Additional reciprocal crosses of the mutants with their wild-type (WT) counterparts separates the female gametophyte (FG)-specific, male gametophyte (MG)-specific, and zygotic mutants from each other (Christensen et al., 1998; Grossniklaus et al., 1998; Howden et al., 1998; Yu et al., 2016) (**Figure 1**).

## Fertilization Recovery System to Recount Fertilization-Defective MG Mutants

Unlike FG, which is sedentary, MG needs to traverse through sporophytic tissues before it reaches to the FG. In addition to MG development, any step from pollen adhesion and germination at the stigma to the pollen tube (PT) burst and successful double fertilization at the FG can be affected by MG mutation thereby bringing a significant variation in the seed-set pattern (Boavida et al., 2009). Numerous MG mutants have been identified by classical approach, which involves screening for the mutants with less-than-normal seed-set followed by the reciprocal crosses with WT and screening for those which show mutation transmission exclusively via MG (Howden et al., 1998; Procissi et al., 2001; Yang et al., 2007; Boavida et al., 2009). However, unlike the FG-specific mutants which show ~50% seed-set, it has been bit tricky to identify the MG mutants from the rate of seed-set alone. The MG mutations affecting the gene/s responsible for either of the pollen development, its competitiveness, PT growth, and its guidance may show a near WT-like (~100%) seed-set when the stigma is oversaturated with mutant derived pollens, provided that the mutant pollens and PTs would not hinder the growth and passage of the normal PT towards FG. However, their subsequent cross with the WT (♀) will show ~50% mutation transmission rate (and ~0% with the reciprocal cross) (Procissi et al., 2001) (**Figure 1**).

When the mutation causes defects in sperm cell (SC) development or function, the self-pollinated hemizygous mutants show a significant drop in seed sets. Few mutants are known to affect SC development, which include *duo1*, *duo2* (Durbarry et al., 2005), *duo3* (Brownfield et al., 2009), *drop1drop2* (Zhang et al., 2017) etc. An additional mutant, *gcs1/hap2* (Mori et al., 2005; von Besser et al., 2006), produces fertilization-defective SCs. Among these mutants, all those tested for seed-set showed 60–75% seed-set rate instead of ~50% (Brownfield et al., 2009; Kasahara et al., 2012). To understand such peculiar seed-set in MG-mutants, we need to understand how a normal FG behaves when the first attempt of fertilization fails. The studies by Kasahara et al. (2012) and Beale et al. (2012) have uncovered the FG behavior at such condition. Normal (WT) FG constitutes two functional synergid cells (SyCs) at its micropylar end. One of those SyCs receive the first PT and degenerate by 10 hours after pollination (HAP) (Kasahara et al., 2012). The SyC which receives PT is referred to as the receptive SyC. In the ovules receiving normal PT, the second SyC (persistent SyC) fuses with the developing endosperm thereby blocking the attraction of any additional PTs (reviewed by Adhikari et al., 2020). However, the persistent SyC of the ovules- receiving mutant (*gcs1/hap2*) PT in the beginning -attracts additional PT to salvage the failed fertilization (Beale et al., 2012; Kasahara et al., 2012). Since the probability of the additional PT being WT is still 50% (when pollinated with *hap2-1/+* pollens), only half of the ovules receiving second PTs may get fertilized and develop into seeds thereby bringing the seed-set rate up to 75% (50% of the ovules fertilized with first PT + 25% of them fertilized with second PT) (**Figure 1**). The proportion of failed-fertilized ovules receiving second PT increases with time from 6% within 10 h after pollination to nearly 40% by 20 HAP (Kasahara et al., 2012). Additionally, about 50% of the failed-fertilized ovules still hold intact persistent SyC by 20 HAP (Beale et al., 2012) in *hap2-1/+* pollinated siliques. However, all failed-fertilized ovules (by the first PT) may not necessarily receive the second PT before they (the ovules) degenerate. This is why the seed-set rate in a fully penetrant fertilization defective hemizygous MG-mutants ranges between 60 and 75% (Brownfield et al., 2009; Kasahara et al., 2012).

It should be noted that any potential mutant with no defect in SC but defective in PT burst may also show ~75% seed-set. The rapid expulsion right after PT burst (Hamamura et al., 2011) is apparently crucial for SCs to reach the egg cell and central cell nuclei followed by their independent fusions during the fertilization process. It should also be noted that, due to the fertilization recovery system at play (Kasahara et al., 2012), a fully penetrant dual gametophytic hemizygous mutant (harboring mutation that affects both MG and FG) may show up to 37.5% seed-set (25% normal ovules fertilized by the first PT + 12.5% normal ovules fertilized by the second PT), but none of its progenies will carry the mutation (**Figure 1**). Additionally, a fully penetrant MG-specific mutant and similar zygotic mutant may show indistinguishable seed-set rates (~75%). Analysis of the marker segregation in their progenies will distinguish the former from the latter as they produce marker positive to negative progenies at 1:1 and 2:1 ratios respectively. (**Figure 1**).

## Linking Vanillin Staining to the Defective Fertilization

Studies show that gametophytic mutations are rare and comprise mere 1–9% of the total mutant pool (Feldmann et al., 1997; Bonhomme et al., 1998; Boavida et al., 2009; Brukhin et al., 2011). Additionally, the majority of the gametophytic mutations show low penetrance and often affect both gametophytes (Bonhomme et al., 1998; Christensen et al., 1998; Grini et al., 1999; Christensen et al., 2002). About 4% of the total mutants may be lost because the affected gene is absolutely essential for both gametes (the population showing 0:1 selection marker segregation at **Figure 1****)**. In such cases, any observed seed-set rate in the self-pollinated hemizygous mutants would be insufficient to determine the gametophytic mutants conclusively.

Classical screening method greatly depends on the selection marker segregation and close linkage of the marker to the mutated gene in the chromosome. Chromosomal rearrangements (and insertions elsewhere in the genome) leading to the development of positive selection marker progenies without mutation in the gene in question is a possible unwanted consequence of the method (Bonhomme et al., 1998). Screening the gametophytic mutants while separating the gametophyte developmental- and fertilization-defective ones without any selection markers would enhance the efficiency and rapidity of the mutant screening process. A recent report by Liu et al. (2020) fills the gap by providing a crucial phenotype-based screening step. This method is relatively quicker and precise as the researchers can determine whether any of the mutations affect fertilization and simultaneously identify the affected gamete type (FG or MG) in a single generation. It exploits the advantage of vanillin staining based on the recent findings of Beale et al. (2012); Kasahara et al. (2016); Liu et al. (2019), and Liu et al. (2020).

**Figure 2:**
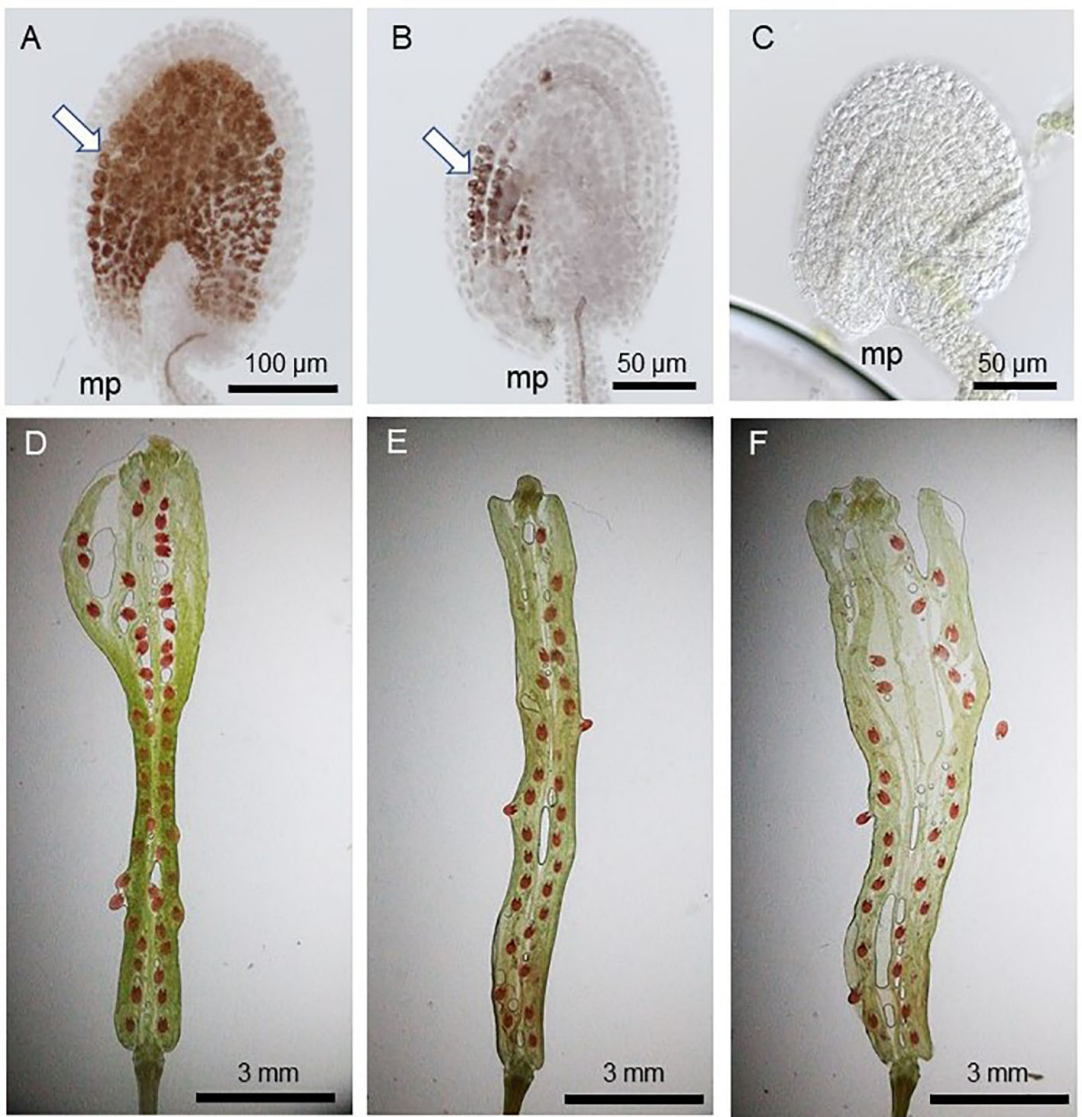
Vanillin staining 3 days after pollination (DAP). **(A)** WT seed with full vanillin staining; **(B)** POEMed ovule with partial vanillin staining; **(C)** not pollinated ovule with no vanillin staining; **(D)** WT silique with ~100% seed-set (1:0:0 full:partial;no vanillin staining); **(E, F)** *gcs1/+* self-pollinated silique with 60–75% seed-set (~3:1:0 full:partial:no vanillin staining); White arrows in **(A, B)** show the vanillin stains. mp = micropyle.

**Figure 3:**
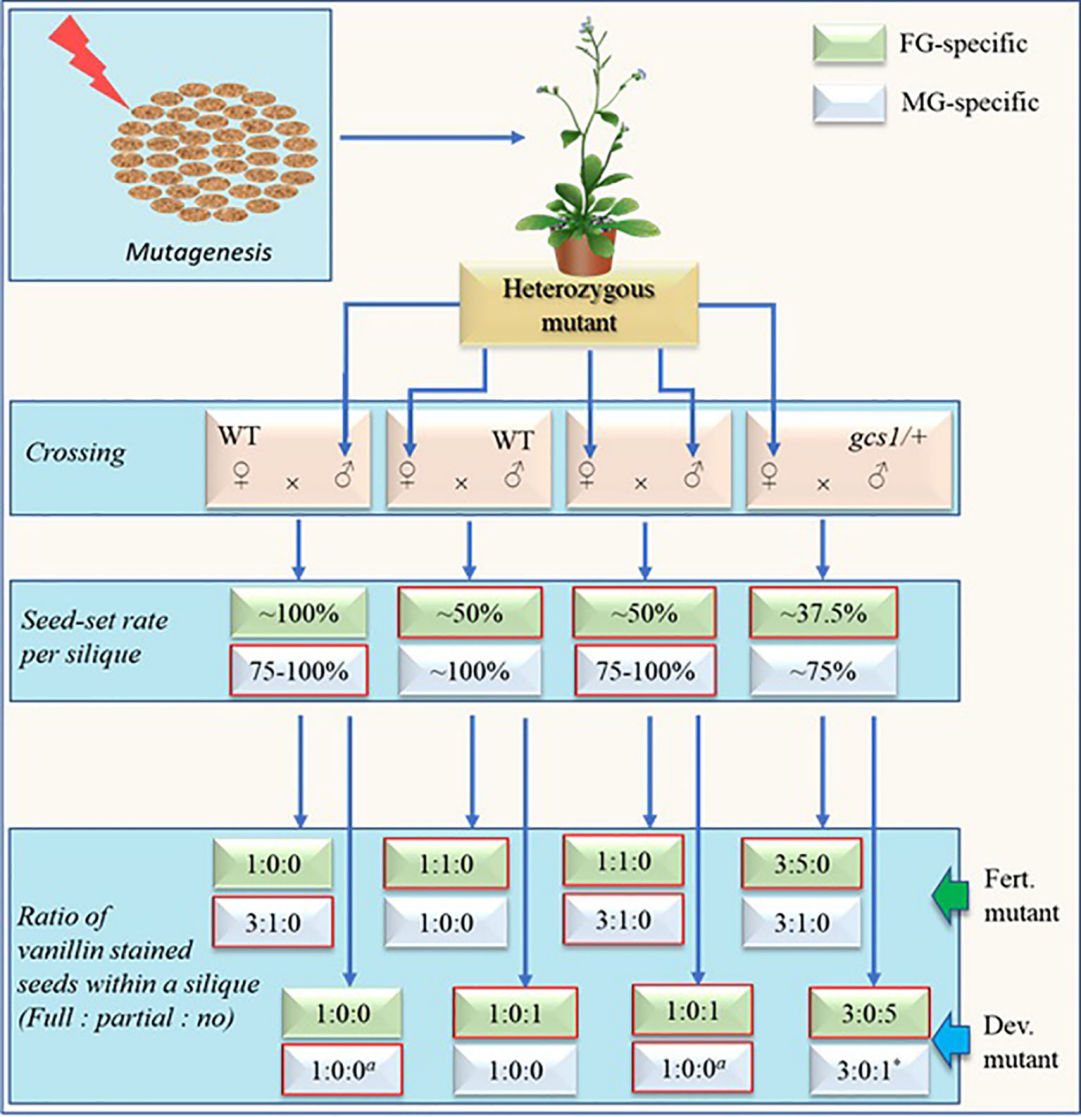
Schematic representation of screening steps (without selection marker assistance) for fully penetrant fertilization defective male or female gametophytic mutants with expected rates and ratios of seed-sets and vanillin staining respectively. Self-cross is sufficient for mutant screening via this approach. Other crosses are shown mainly for the comparison purpose. Developmental-defective mutants (dev. Mutants) have defects in the development of either female gametophyte (FG) or vegetative cell of male gametophyte (MG) while fertilization-defective mutants (fert. Mutants) have defects either in FG at fertilization step or MG during the development and/or function of sperm cells (SCs). The seed-setting rate will remain ~75% in SC-defective MG mutants while it may increase to near 100% in vegetative-cell-defective mutants. The numerical figures represent the theoretically possible maximum values determined largely based on the studies by Kasahara et al. (2012) and Liu et al. (2020). Expected rates and ratios for respective mutants are in boxes with red margin. *a* = The values depend on the degree by which the stigma is saturated with pollens. The displayed values represent the case when the stigma is excessively pollinated. Upon limited pollination, the number of full/partial-stained ovules may decrease while that of unstained ovules may increase. * = The value will remain true only when the pollen donor MG mutant is defective in pollen development, pollen tube (PT) growth and/or PT-burst at the ovule instead of SC-defective *gcs1/+*.

Since pollen tube content (PTC) release in the ovule triggers seed coat formation and the successful fertilization allows its continued and complete formation (Liu et al., 2019), the fertilized ovules show full vanillin staining (**Figure 2A**) while those which received PTC but failed fertilization show partial staining (**Figure 2B**). The ovules lacking PTC (and fertilization), on the other hand, do not stain at all (**Figure 2C**). **Figures 2D–F** show varied rates of seed-setting and ratios of vanillin staining in WT and *gcs1/+* mutants, as explained in **Figure 3**.

## Empirical Definition of Vanillin Based Gametophytic Mutant Screening

Expected maximum seed-set rates and proportions of vanillin staining (full:partial:no) within a silique of either developmental- or fertilization-defective MG and/or FG-mutants are shown in **Figure 3**. Two simultaneous steps of this new screening method include: *1)* Carrying out self-cross and check the seed-set pattern in the mutants and *2)* using the siliques of the mutants for vanillin staining 3 days after pollination and check the proportion of fully stained, partially stained, and unstained ovules. The fertilization defective mutants will show no unstained ovules while the developmental-defective mutants will show no partially stained ovules. Additional crosses (other than the self-cross) shown in **Figure 3** are for the comparison purpose (with self-cross derived empirical values). Additionally, the values of the cross with *gcs1/+* (♂) in **Figure 3** remain true for the self-crossed fully penetrant dual-gametophytic hemizygous mutants as well.

A fertilization-defective FG mutant will show ~50% seed-set upon self-cross (or crossing with WT (♂)). The vanillin staining will exhibit the ratio of 1:1:0 (full:partial:no) for the ovules in each silique. Additionally, its cross with *gcs1/+* (♂) (a cross equivalent to the self-cross of a fully penetrant fertilization-defective dual-gametophytic mutant) would show 37.5% seed-set. The vanillin staining would show the ratio of 3:5:0 (full:partial:no), with ~37.5 and ~62.5% seeds/ovules showing full and partial staining, respectively (Liu et al., 2019). Hence, this screening approach may identify those ~4% of the mutants affecting both gametes, which would otherwise be lost during the classical screening process (Bonhomme et al., 1998). The ~37.5% seed-set and 3:5:0 (or 3:0:1 when MG is developmental-defective) vanillin staining ratio in a mutant is a sign for the researcher to save it and analyze before it is lost.

A fertilization-defective MG mutant, on the other hand, will show ~75% seed-set upon self-cross or crossing with WT (♀). Subsequent vanillin staining would show the ratio of 3:1:0 (full:partial:no) for the ovules in each silique (**Figure 3**). As discussed in above section, the seed-set rate of a MG mutant- defective in any step from pollen development to PT burst (in the FG) -may vary with the efficiency of its PTs reaching to the ovules, and releasing PTC (along with the SCs). Some of these mutants may show WT-like seed-set and escape during the screening process. However, they can be distinguished by conducting a comparative time series silique observation. These mutants are likely to show higher PT density at TT and/or delayed fertilization as compared to their WT counterparts.

It should be noted that the seed-set rates and vanillin staining proportions shown in **Figure 3** are likely to skew significantly for the less penetrant mutants. Additionally, this screening method may not separate the MG-mutants from the zygotic mutants in a single generation since both mutants may show similar seed-set rates (~75%) and vanillin staining ratios (3:1:0) (**Figures 1** and **3**). It can be circumvented by crossing the mutants (♂) with the WT (♀) since it would show WT-like (~100%) or ~75% seed-set for the zygotic or fertilization-defective-MG mutants respectively.

## Conclusion

Vanillin staining combined with the consideration of the fertilization recovery system offers a strong clue for screening gametophytic mutants (except for the fertilization-defective MG-mutants vs zygotic mutants) in a single generation. When selection marker segregation analysis is added (one additional generation), this screening approach can effectively separate any of the zygotic and fertilization-/developmental-defective MG/FG mutants. Moreover, starting the screening process (after mutagenesis) from vanillin-staining of the self-crossed mutant siliques provides a rough clue on the mutant type (MG/FG-specific fertilization/developmental mutants) and allows a researcher to prepare a gametophytic mutant enriched pool for their targeted gametophytic studies.

## Author Contributions

PA prepared the data figures and wrote the manuscript. XL carried out the vanillin staining experiments, and RK checked and confirmed the data and manuscript.

## Funding

This work was supported by start-up funds from the School of Life Sciences, Fujian Agriculture and Forestry University (Grant #: 114-712018008 to RK) and the FAFU-UCR Joint Center, Haixia Institute of Science and Technology, Fujian Agriculture and Forestry University. This work was also supported by Chinese NSFC fund (Grant #: 31970809). This work was also supported by the Precursory Research for Embryonic Science and Technology (Grant #: 13416724 to RK; Kasahara Sakigake Project, Japan Science and Technology Agency).

## Conflict of Interest

The authors declare that the research was conducted in the absence of any commercial or financial relationships that could be construed as a potential conflict of interest.

## References

[B1] Adhikari P. B. Liu X. Wu X. Zhu S. Kasahara R. D. (2020). Fertilization in flowering plants: an odyssey of sperm cell delivery. Plant Mol. Biol. 103 (1–2), 9–32. 10.1007/s11103-020-00987-z 32124177

[B2] Beale K. M. Leydon A. R. Johnson M. A. (2012). Gamete fusion is required to block multiple pollen tubes from entering an *Arabidopsis* ovule. Curr. Biol. 22 (12), 1090–1094. 10.1016/j.cub.2012.04.041 22608506 PMC3973743

[B3] Boavida L. C. Shuai B. Yu H.-J. Pagnussat G. C. Sundaresan V. McCormick S. (2009). A collection of *Ds* insertional mutants associated with defects in male gametophyte development and function in *Arabidopsis thaliana*. Genetics 181 (4), 1369–1385. 10.1534/genetics.108.090852 19237690 PMC2666506

[B4] Bonhomme S. Horlow C. Vezon D. de Laissardière S. Guyon A. Férault M. (1998). T-DNA mediated disruption of essential gametophytic genes in Arabidopsis is unexpectedly rare and cannot be inferred from segregation distortion alone. Mol. Gen. Genet. 260 (5), 444–452. 10.1007/s004380050915 9894914

[B5] Brownfield L. Hafidh S. Durbarry A. Khatab H. Sidorova A. Doerner P. (2009). *Arabidopsis* DUO POLLEN3 is a key regulator of male germline development and embryogenesis. Plant Cell 21 (7), 1940–1956. 10.1105/tpc.109.066373 19638475 PMC2729611

[B6] Brukhin V. B. Jaciubek M. Carpio A. B. Kuzmina V. Grossniklaus U. (2011). Female gametophytic mutants of *Arabidopsis thaliana* identified in a gene trap insertional mutagenesis screen. Int. J. Dev. Biol. 55 (1), 73–84. 10.1387/ijdb.092989vb 21425082

[B7] Christensen C. A. Subramanian S. Drews G. N. (1998). Identification of gametophytic mutations affecting female gametophyte development in *Arabidopsis*. Dev. Biol. 202 (1), 136–151. 10.1006/dbio.1998.8980 9758709

[B8] Christensen C. A. Gorsich S. W. Brown R. H. Jones L. G. Brown J. Shaw J. M. (2002). Mitochondrial GFA2 is required for synergid cell death in Arabidopsis. Plant Cell 14 (9), 2215–2232. 10.1105/tpc.002170 12215516 PMC150766

[B9] Durbarry A. Vizir I. Twell D. (2005). Male germ line development in Arabidopsis. *duo pollen* mutants reveal gametophytic regulators of generative cell cycle progression. Plant Physiol. 137 (1), 297–307. 10.1104/pp.104.053165 15618418 PMC548860

[B10] Feldmann K. A. Coury D. A. Christianson M. L. (1997). Exceptional segregation of a selectable marker (KanR) in Arabidopsis identifies genes important for gametophytic growth and development. Genetics 147 (3), 1411–1422. 9383081 10.1093/genetics/147.3.1411 PMC1208262

[B11] Grini P. E. Schnittger A. Schwarz H. Zimmermann I. Schwab B. Jürgens G. (1999). Isolation of ethyl methanesulfonate-induced gametophytic mutants in *Arabidopsis thaliana* by a segregation distortion assay using the multimarker chromosome 1. Genetics 151 (2), 849–863. 9927475 10.1093/genetics/151.2.849 PMC1460497

[B12] Grossniklaus U. Vielle-Calzada J.-P. Hoeppner M. A. Gagliano W. B. (1998). Maternal control of embryogenesis by *MEDEA a Polycomb* group gene in *Arabidopsis*. Science 280 (5362), 446–450. 10.1126/science.280.5362.446 9545225

[B13] Hamamura Y. Saito C. Awai C. Kurihara D. Miyawaki A. Nakagawa T. (2011). Live-cell imaging reveals the dynamics of two sperm cells during double fertilization in *Arabidopsis thaliana*. Curr. Biol. 21 (6), 497–502. 10.1016/j.cub.2011.02.013 21396821

[B14] Howden R. Park S. K. Moore J. M. Orme J. Grossniklaus U. Twell D. (1998). Selection of T-DNA-tagged male and female gametophytic mutants by segregation distortion in Arabidopsis. Genetics 149 (2), 621–631. 9611178 10.1093/genetics/149.2.621 PMC1460162

[B15] Hsieh T.-F. Shin J. Uzawa R. Silva P. Cohen S. Bauer M. J. (2011). Regulation of imprinted gene expression in *Arabidopsis* endosperm. Proc. Natl. Acad. Sci. 108 (5), 1755–1762. 10.1073/pnas.1019273108 21257907 PMC3033266

[B16] Kasahara R. D. Maruyama D. Hamamura Y. Sakakibara T. Twell D. Higashiyama T. (2012). Fertilization recovery after defective sperm cell release in *Arabidopsis*. Curr. Biol. 22 (12), 1084–1089. 10.1016/j.cub.2012.03.069 22608509

[B17] Kasahara R. D. Notaguchi M. Nagahara S. Suzuki T. Susaki D. Honma Y. (2016). Pollen tube contents initiate ovule enlargement and enhance seed coat development without fertilization. Sci. Adv. 2 (10), e1600554. 10.1126/sciadv.1600554 27819041 PMC5091356

[B18] Liu X. Adhikari P. B. Kasahara R. D. (2019). Pollen tube contents from failed fertilization contribute to seed coat initiation in *Arabidopsis*. F1000Research 8 (348), 348. 10.12688/f1000research.18644.2 31031972 PMC6468697

[B19] Liu X. Wu X. Adhikari P. B. Zhu S. Kinoshita Y. Berger F. (2020). Establishment of a novel method for the identification of fertilization defective mutants in *Arabidopsis thaliana*. Biochem. Biophys. Res. Commun. 521 (4), 928–932. 10.1016/j.bbrc.2019.11.028 31711640

[B20] Mori T. Kuroiwa H. Higashiyama T. Kuroiwa T. (2005). GENERATIVE CELL SPECIFIC 1 is essential for angiosperm fertilization. Nat. Cell Biol. 8, 64. 10.1038/ncb1345 16378100

[B21] Procissi A. de Laissardiere S. Ferault M. Vezon D. Pelletier G. Bonhomme S. (2001). Five gametophytic mutations affecting pollen development and pollen tube growth in *Arabidopsis thaliana*. Genetics 158 (4), 1773–1783. 11514461 10.1093/genetics/158.4.1773 PMC1461763

[B22] Satyaki P. R. V. Gehring M. (2017). DNA methylation and imprinting in plants: machinery and mechanisms. Crit. Rev. Biochem. Mol. Biol. 52 (2), 163–175. 10.1080/10409238.2017.1279119 28118754

[B23] Vivian-Smith A. Luo M. Chaudhury A. Koltunow A. (2001). Fruit development is actively restricted in the absence of fertilization in *Arabidopsis*. Development 128 (12), 2321–2331. 11493551 10.1242/dev.128.12.2321

[B24] von Besser K. Frank A. C. Johnson M. A. Preuss D. (2006). *Arabidopsis HAP2* (*GCS1*) is a sperm-specific gene required for pollen tube guidance and fertilization. Development 133 (23), 4761–4769. 10.1242/dev.02683 17079265

[B25] Yang H. Li W. Chen S. (2007). Isolation and characterization of 4 gametophytic male sterile mutants in *Arabidopsis thaliana*. Chin. Sci. Bull. 52 (14), 1949–1956. 10.1007/s11434-007-0279-9

[B26] Yu T. Y. Shi D. Q. Jia P. F. Tang J. Li H. J. Liu J. (2016). The Arabidopsis receptor kinase ZAR1 is required for zygote asymmetric division and its daughter cell fate. PloS Genet. 12 (3), e1005933–e1005933. 10.1371/journal.pgen.1005933 27014878 PMC4807781

[B27] Zhang J. Huang Q. Zhong S. Bleckmann A. Huang J. Guo X. (2017). Sperm cells are passive cargo of the pollen tube in plant fertilization. Nat. Plants 3, 17079–17079. 10.1038/nplants.2017.79 28585562 PMC5960590

